# In-Depth Insight into the Effects of Steel Slag and Calcium Hydroxide on the Properties of a Fly Ash–Red Mud Geopolymer

**DOI:** 10.3390/ma17061249

**Published:** 2024-03-08

**Authors:** Penghuai Wang, Ping Chen, Yang Ming, Qing Li, Xuanxuan Dong

**Affiliations:** 1 College of Materials Science and Engineering, Guilin University of Technology, Guilin 541004, China; 2 Guangxi Key Laboratory of Green Building Materials and Construction, Guilin University of Technology, Guilin 541004, China; 3 Collaborative Innovation Center for Exploration of Nonferrous Metal Deposits and Efficient Utilization of Resources, Guilin University of Technology, Guilin 541004, China; 4 Guangxi Engineering and Technology Center for Utilization of Industrial Waste Residue in Building Materials, Guilin University of Technology, Guilin 541004, China

**Keywords:** geopolymer, compressive strength, hydration products, calcium hydroxide

## Abstract

The low mechanical strength of a low-calcium fly ash (FA)–red mud (RM) geopolymer severely limits its application. Steel slag (SS) and Ca(OH)_2_ can provide calcium and alkali for the hydration process of a low-calcium FA-based geopolymer. In this study, SS was used to replace part of the RM, and Ca(OH)_2_ was introduced. The effects of SS and Ca(OH)_2_ on the properties of the FA-RM geopolymer were investigated. The experimental results show that SS promoted the matrix to generate more C(N)-A-S-H and C-S-H gels and optimized the pore structure, thereby improving the mechanical properties of the FA-based geopolymer. The addition of 4 wt.% Ca(OH)_2_ increased the hydration products of the FA-based geopolymer, the microstructure was denser, and the mechanical properties were significantly improved. The 28 d compressive strength of the FA-based ternary composite geopolymer prepared by replacing part of the RM with SS and adding Ca(OH)_2_ reached 30.6 MPa, which provided an experimental basis for the resource utilization of various bulk solid wastes.

## 1. Introduction

With the rapid development of metallurgy, mining, and chemical industries, the emission of industrial solid waste is increasing day by day. Among them, fly ash (FA), steel slag (SS), and red mud (RM), as China’s bulk industrial solid wastes, have a low utilization rate [1]. They not only occupy a large amount of land when stacked in the open air but also cause dust to pollute the atmosphere. With the erosion of rainwater and the passage of time, they will also pollute water resources and destroy the acid-base balance of soil, which will bring a series of environmental problems and affect economic development [2]. Therefore, if we can make use of the characteristics of these solid wastes to prepare a building material and dispose of these solid wastes while giving full play to their surplus value, it will have significant economic and social benefits.

Geopolymers are a family of aluminosilicates synthesized by alkali-activating Si- and Al-rich materials and polycondensing tetrahedral silica and alumina [3]. As an emerging class of cementitious materials, geopolymers have some noteworthy characteristics, such as exceptional mechanical strength, resistance to corrosion and water, the ability to withstand high temperatures, and immobilization of metal ions [4]. The raw materials for preparing geopolymers are abundant. Generally, any aluminosilicate material with potential volcanic ash properties contains amorphous SiO_2_ and Al_2_O_3_ components and can be used as a raw material for geopolymers [5,6].

FA is an industrial waste produced by coal-fired power plants. Because FA contains a large amount of highly active silicates and aluminates, it is the most common material used to prepare geopolymers [7]. RM, also known as bauxite slag, is an industrial waste residue after extracting alumina from bauxite by the Bayer method [8]. RM is used to prepare geopolymers by mixing with other silicon-rich and aluminum-rich materials because of its strong alkalinity, thereby reducing the consumption of alkali activators to produce additional economic benefits, and alkali activators are the most expensive raw materials required for geopolymer synthesis [9,10]. In recent years, the preparation of geopolymers with FA and RM as raw materials has received extensive attention. Zhang et al. prepared RM-FA-based geopolymers at room temperature and studied the factors affecting mechanical properties, microstructure, and chemical composition. It was found that an increase in Si/Al and Na/Al ratios would lead to an increase in geopolymer strength [3]. Manish Mudgal et al. proved that the geopolymer prepared from FA and RM (FA-RM-geopolymer) has a significantly high strength of 65 MPa under optimized experimental conditions [11]. However, the low-calcium FA-based geopolymer solidified at room temperature has the disadvantages of slow setting and hardening, low early strength, and a long curing cycle, which limit its application [12,13]. Hu et al. studied the preparation of geopolymers with RM and three different FAs. It was found that the geopolymer synthesized with RM and C-grade FA at room temperature obtained a compressive strength of 15.2 MPa, while the geopolymer prepared with RM and F-grade FA could not obtain the available strength [14]. This is because the disadvantage of alkali-activated materials in low-calcium systems is that the early strength is low, and proper ‘calcium supplementation’ in low-calcium systems can overcome this disadvantage well [15]. Many scholars expect to prepare FA-based composite geopolymer materials with excellent mechanical properties at room temperature by introducing other calcium-rich materials into a low-calcium FA-based geopolymer [16].

SS is a major waste residue in the iron and steel industry. Due to its large fluctuations in chemical composition and poor activity, its utilization rate is much lower than other solid wastes, but its calcium content is high and can be used as a calcium source for low-calcium geopolymer systems [17]. As Jiang Yong et al. delved into the formulation of alkali-activated FA-SS cementitious materials, they noted that the incorporation of SS stimulated the formation of greater quantities of sodium-based smithsonite within the system. This phenomenon notably bolstered the strength of the cementitious materials [18]. I. Niklioća et al. also found that the addition of SS increased the strength of FA-based geopolymer [19].

In addition, as far as the author knows, high-temperature curing or increasing alkalinity has been proven to be a feasible method to improve the strength of FA-based geopolymers, but high-temperature curing may hinder the application of geopolymers as building materials [20]. Therefore, in this paper, aiming at the problem of low mechanical strength of the FA-RM geopolymer, a FA-SS-RM ternary geopolymer material was prepared by replacing part of the RM with SS, and the strength was improved by introducing Ca(OH)_2_ to increase the alkalinity and calcium content of the system. The effects of SS and Ca(OH)_2_ on the matrix strength, hydration products, and microstructure of the FA-RM-based geopolymer were studied.

## 2. Materials and Methods

### 2.1. Materials

The FA came from the Baotou Iron and Steel Group in Baotou City, China, and was F grade, with a density of 2.34 g/cm^3^ and a specific surface area of 530 m^2^/kg. The Bayer RM came from Pingguo Aluminum Co., Ltd., Baise City, China. The density is 3.11 g/cm^3^, and the specific surface area is 670 m^2^/kg after ball milling. The converter SS was obtained from Liuzhou Iron and Steel Company in Liuzhou City, China, with a density of 3.26 g/cm^3^, fined to 600 mesh by ball mill, and a specific surface area of 499 m^2^/kg. The sand was the standard sand produced by Xiamen ISO Standard Sand Co., Ltd., Xiamen, China. NaOH, Ca(OH)_2,_ and sodium silicate were produced by Xilong Science Co., Ltd., (Shantou, China) in which NaOH and Ca(OH)_2_ were flakes, analytical reagent, the solid content of sodium silicate was 45.16% (mass fraction), and the modulus was 3.26.

### 2.2. Sample Preparation

Due to the huge production, serious harm, and difficult disposal task of the RM in the Guangxi Province of China, it is expected that researchers use the red mud as much as possible during the preparation of a geopolymer. According to the previous experimental exploration, the FA-RM geopolymer prepared when the mass ratio of FA to RM is 1:1 has relatively high mechanical properties and the largest amount of RM used. Similarly, the FA-SS-RM geopolymer prepared when the mass ratio of FA:SS:RM is 2:1:1 also has relatively high mechanical properties and the largest amount of RM used. The alkali activator modulus used in the sample preparation is 1.5, the alkali equivalent (calculated by Na_2_O) is 6% of the cementitious material, the water–binder ratio is 0.35, and the Ca(OH)_2_ content is 4%. FA, SS, RM, Ca(OH)_2,_ and sand were mixed in a cement mixer according to the ratio. Then, the solution prepared with sodium silicate, NaOH, and water was poured into the mixer and stirred for 2 min. Then, the obtained mixture was poured into a test mold with a size of 40 mm × 40 mm × 160 mm for vibration molding (the paste was poured into a 40 mm × 40 mm × 40 mm test mold for the same treatment). Then, the mold was covered with plastic wrap and placed in a curing room with a temperature of (20 ± 2) °C and a relative humidity of more than 95%. After 24 h of curing, the mold was demolded and then continued to be cured in the curing room to the age. The mix proportions of mortars are shown in Table 1. When the paste specimen is formed, the fine aggregate is removed, and the proportion of other materials is kept unchanged.

### 2.3. Test Methods

The compressive strength test was carried out according to GB/T 17671-2021 “Cement mortar strength test method (ISO method)” [21]. The EDX-LE Plus X-ray fluorescence spectrometer (SHIMADZU CORPORATION, Kyoto, Japan) was used to analyze the chemical composition of the raw materials. The phase composition of hydration products was analyzed by X-ray diffractometer (PANantical X′ Pert PRO X, PANalytical B.V. Amsterdam, The Netherlands), (Cu Kα1 target, diffraction angle 5–~80°, continuous scanning, scanning speed 10°/min). The bonding position of hydration products were analyzed by Nicolet 5DXC Fourier transform infrared spectrometer (Nicolet, Madison, WI, USA), (KBr pellet method, resolution of 2 cm^−1^, scanning range of 400–4000 cm^−1^). The mass loss (TG-DTG) of hydration products at different temperatures was measured by STA 449 F5 Jupiter comprehensive thermal analyzer (NETZSCH, Selb, Germany). The temperature range was 20–1000 °C, the heating rate was 10 °C/min, and the test atmosphere was nitrogen. The porosity of the sample was analyzed by an automatic mercury porosimeter AutoPore V 9600 (Micromeritics, Shanghai, China). The maximum mercury injection pressure of the test parameters was 414 MPa, and the pore size test range was 3 nm–800 μm. The microstructure of hydration products was characterized by Zeiss Gemini SEM300 scanning electron microscope (SEM), and the elemental composition of hydration products was determined by an X-ray energy spectrometer (Carl Zeiss AG, Oberkochen, Germany). 

## 3. Results and Discussion

### 3.1. Raw Material Analysis

**Figure 1:**
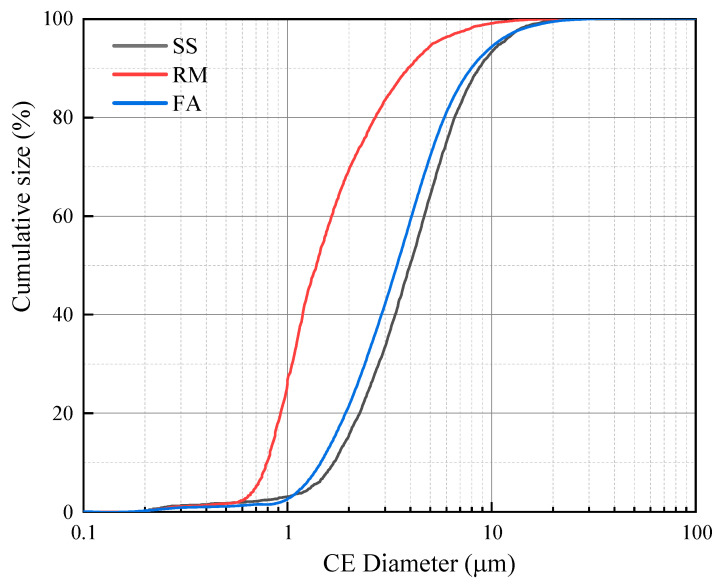
Particle size distribution of raw materials. (SS: steel slag, RM: red mud, FA: fly ash).

**Figure 2:**
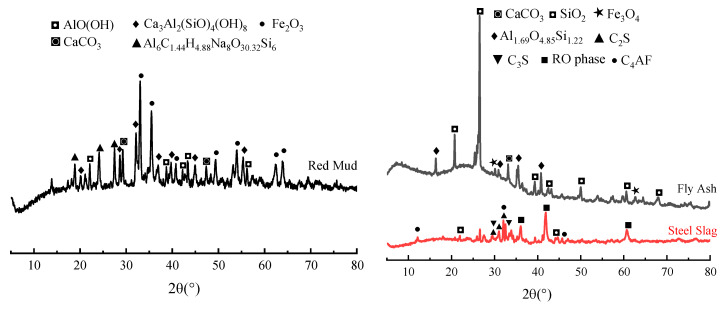
XRD patterns of raw materials.

The particle size distribution of the RM is significantly smaller than that of the FA and SS (Figure 1). Figure 2 is the XRD pattern of raw materials, and Table 2 provides the main chemical composition of raw materials. The main mineral phases in SS are C2S, RO, and C4AF, which can provide sufficient calcium sources. The content of Na_2_O in the RM is 9.15%, which provides part of OH^−^ for the dissolution of precursor silicon and aluminum. At the same time, silicon and aluminum in the RM can also participate in the hydration reaction of the matrix.

### 3.2. Mechanical Property Analysis

**Figure 3:**
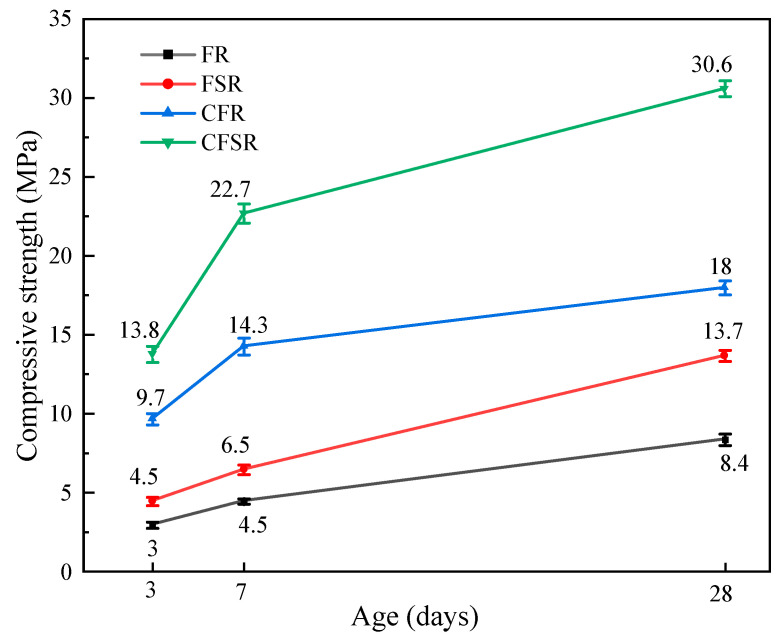
Compression strength of different samples.

The compressive strength of the sample FSR at each age is higher than that of the sample FR. The compressive strength of the sample CFSR at each age is also much larger than that of the sample CFR (Figure 3). It shows that the addition of SS significantly improves the compressive strength of the geopolymer whether or not Ca(OH)_2_ is added. One possible explanation is that the soluble aluminosilicate in SS is higher than that in RM, and the alkaline activator, the alkali in RM, and the added Ca(OH)_2_ can improve the reactivity of the geopolymer system by dissolving the aluminosilicate in SS, forming more silicon–oxygen tetrahedrons and aluminum–oxygen tetrahedrons [22]. In addition, Sun et al. also found that alkali-activated SS has similar hydration processes and products to cement [23]. The addition of SS can significantly improve the hydration activity of the geopolymer system and promote the formation of more hydration products such as C-S-H gel in the matrix [24].

Another significant phenomenon in the experiment is that the compressive strength of the geopolymer increases significantly after the addition of Ca(OH)_2_. The specific performance is that the 28-day compressive strength of the sample CFR is 114.3% higher than that of the sample FR, and the 28-day strength of the sample CFSR is 123.4% higher than that of the sample FSR. This is because the increase in the alkalinity of the geopolymer system after the addition of Ca(OH)_2_ makes the soluble aluminosilicate in the precursor completely dissolve. At the same time, Ca(OH)_2_ itself also introduces a part of Ca^2+^ as the calcium source of the system, and the higher ion concentration reacts to generate more gel substances, which improves the compactness of the matrix. Temuujin et al.’s research also shows that Ca(OH)_2_ can promote the formation of C-A-S-H in FA-based geopolymers and greatly improve the early strength [25]. In summary, the use of SS and Ca(OH)_2_ can greatly improve the compressive strength of the FA-RM geopolymer matrix, and the prepared FA-SS-RM composite geopolymer material has good mechanical properties.

In addition, we also found that the early compressive strength rate of sample FR and sample FSR is relatively slow, and the growth rate from 3 d to 7 d is similar to the growth rate from 7 d to 28 d. However, the early strength of CFR and CFSR samples doped with Ca(OH)_2_ has a large growth rate, and the growth rate from 3 d to 7 d is much larger than that from 7 d to 28 d. This explains why Ca(OH)_2_ accelerates the dissolution of the precursor and the formation of the geopolymer.

### 3.3. XRD Analysis

**Figure 4:**
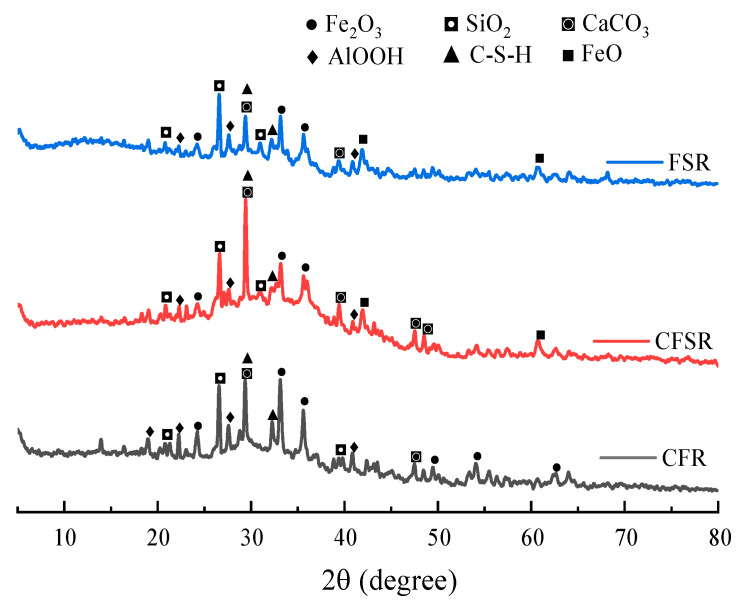
XRD pattern of the samples cured for 28 d.

The XRD diffraction peaks of hydration products of the three samples are similar (Figure 4), mainly including hematite (Fe_2_O_3_), quartz (SiO_2_), calcite (CaCO_3_), and C-S-H. Notably, the main hydration products of geopolymer specimens are amorphous C-S-H and C(N)-A-S-H gels with a low-crystalline torrefied mullite structure, which corresponded to characteristic peaks that were not obvious in the XRD plots [26]. It can be seen that the peak intensity of C-S-H in the XRD pattern of the sample CFSR and CFR are larger than those of the sample FSR. This is because the incorporation of Ca(OH)_2_ increases the content of Ca(OH)_2_, OH^−^, and Ca^2+^ in the system, and the pH of the system becomes larger, prompting the precursor to dissolve more silicon–aluminum substances, and these silicon–aluminum substances will react with Ca(OH)_2_ to form C(N)-A-S-H and C-S-H gels [27]. Meanwhile, the peak intensity of C-S-H increased after the incorporation of SS, indicating that the addition of SS can promote the formation of more gels in the matrix, which also explains why the compressive strength of the sample CFSR is much larger than that of the sample CFR.

### 3.4. FTIR Analysis

**Figure 5:**
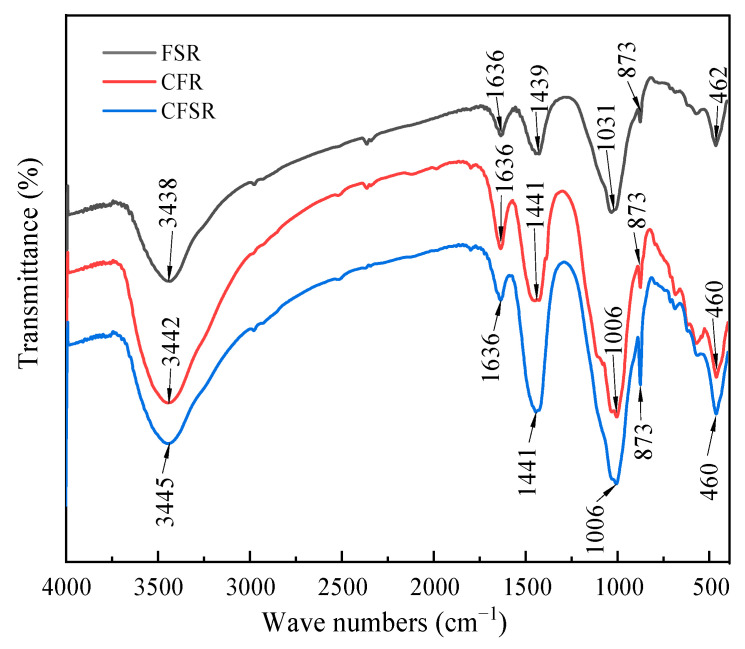
FTIR patterns of the sample cured for 28 d.

XRD can only characterize the crystalline or semi-crystalline components, and the samples were further analyzed by FTIR. The FTIR spectra of different samples are very similar and have the same absorption bands (Figure 5). The absorption peaks near 3442 cm^−1^ and 1636 cm^−1^ are related to the stretching vibration of the O-H bond in the hydration products [28]. The characteristic peaks near 1440 cm^−1^ and 870 cm^−1^ are caused by the asymmetric stretching vibration of CO_3_^2−^, corresponding to the carbonation reaction of hydration products [29]. About 1006 cm^−1^ and 460 cm^−1^ are the characteristic peaks caused by the asymmetric stretching vibration of the (Al, Si)-O-Si bond, and combined with the results of the XRD pattern, we can attribute these characteristic peaks to the C(N)-A-S-H and C-S-H gels in the hydration products [30,31]. The peak shapes of the sample CFR and CFSR vibration peaks with wave numbers of about 1006 cm^−1^ and 460 cm^−1^ are sharper and larger than those of the sample FSR. This is related to the high Ca content in CFR and CFSR samples, which helps to improve the solubility of the precursor [32]. In addition, the peak around 1031 cm^−1^ was shifted to low frequency which was around 1006 cm^−1^ after adding Ca(OH)_2_. This is due to the incorporation of Ca(OH)_2_; there is more inorganic gel product Si-O-Al chain formation, resulting in the local chemical environment of (Al, Si)-O-Si bond changing and shifting to the low wave-number direction [33]. Finally, it can be seen that the vibration peak area of the sample CFSR with a wave number of about 1006 cm^−1^ and 460 cm^−1^ is larger than that of the sample CFR, indicating that the incorporation of SS can also promote the formation of gel products.

### 3.5. TG-DTG Analysis

**Figure 6:**
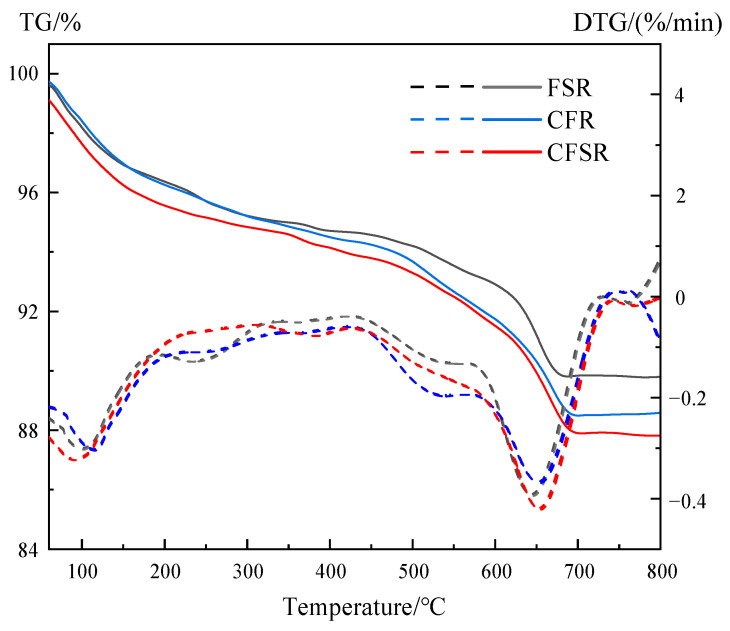
TG-DTG curves of the samples at 28 d.

It can be seen that the four samples have similar weight loss trends and similar endothermic peaks (Figure 6). Combined with the results of XRD and FTIR analysis, it can be seen that the endothermic peak of the DTG curve in the temperature range of 60–300 °C is related to the hydration products C(N)-A-S-H and C-S-H, the endothermic peak in the temperature range of 450–550 °C is related to the decomposition of Ca(OH)_2_, and the endothermic peak in the temperature range of 600–700 °C is attributed to the decomposition of CaCO_3_ [34,35].

The mass loss of the TG curve in the temperature range of 60–600 °C is mainly due to the evaporation of free water and the removal of bound water in C(N)-A-S-H and C-S-H gels. It can be seen from Figure 6 that the mass loss of the sample FSR is the smallest in the temperature range of 60–600 °C and is much smaller than that of the sample CFSR, indicating that the addition of Ca(OH)_2_ can significantly increase the formation of C(N)-A-S-H and C-S-H gels, thereby greatly improving the strength. In addition, the mass loss of the sample CFSR in this temperature range is slightly larger than that of the sample CFS, which also indicates that the incorporation of SS can promote the formation of a gel, which is consistent with the previous FTIR analysis results.

### 3.6. Mercury Intrusion Porosimetry (MIP) Analysis

The mechanical properties of the matrix are closely related to its internal pore structure [36]. The pore size distribution includes small pores (≤20 nm), mesopores (20–50 nm), and macropores (≥50 nm), among which macropores are harmful to the mechanical properties of the matrix [37].

**Figure 7:**
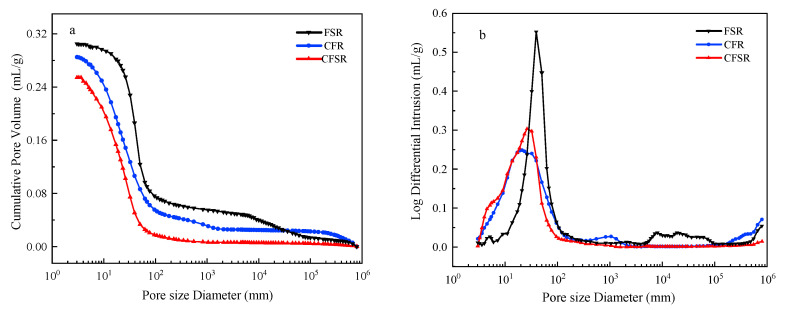
(**a**) Cumulative pore volume and (**b**) log differential invasion curve.

The cumulative pore volume, average pore diameter, and porosity of CFR and CFSR samples doped with Ca(OH)_2_ are lower than those of FSR samples without Ca(OH)_2_, which is related to the volume density of the samples (Figure 7 and Table 3). Combined with the previous analysis results, it is believed that the addition of Ca(OH)_2_ can make the samples produce more hydration products, increase the volume density of the matrix, and reduce the internal porosity. Comparing the pore size distribution data of CFSR samples and FSR samples, the proportion of small pores and macropores in the sample FSR is 10.63% and 40.58%, respectively. In comparison, the proportion of small pores and macropores in the sample CFSR is 48.67% and 13.15%, respectively. The data show that more hydration products can fill the large pores of the matrix and convert them into small pores to improve the compactness and to improve the internal structure and mechanical properties of the matrix.

Furthermore, we can also obtain the information that the sample CFSR has lower porosity and a less harmful pore proportion than the sample CFR. Combined with the other pore structure parameters of the two samples and the previous analysis results, a possible explanation is that on the one hand, the sample CFSR generates more hydration products than the sample CFR to fill the harmful pores in the matrix, and on the other hand, the incorporation of SS has different particle size gradations with the original FA and RM particles. The close packing between these particles and hydration products can also reduce the proportion of harmful pores in the matrix [27].

### 3.7. SEM-EDS Analysis

**Figure 8:**
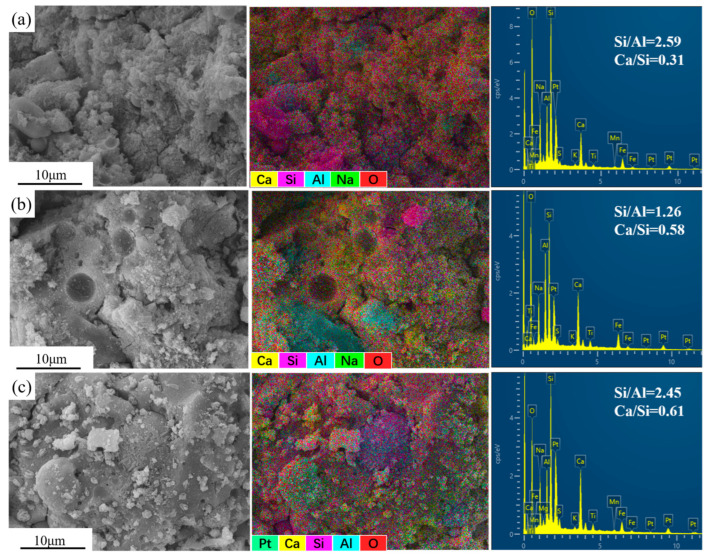
SEM-EDS images of samples ((**a**): FSR, (**b**): CFR, (**c**): CFSR).

Figure 8 shows the SEM images, the SEM-EDS elemental mapping, and the EDS spectrum of different samples after 28 days of curing. It can be seen from the SEM-EDS elemental mapping that the main components of the hydration products of the three samples are similar, all of which are Ca, Si, Al, Na, and O, which also indicates that the main hydration products of the three samples are C(N)-A-S-H and C-S-H gels. However, the element ratio of different samples is different. The sample FSR has the lowest Ca/Si ratio, only 0.31. In contrast, the sample CFSR with the same precursor composition increases to 0.61 due to the addition of 4% Ca(OH)_2_, indicating that Ca(OH)_2_ changes the performance and content of C(N)-A-S-H and C-S-H [38]. In addition, it can be seen that the sample CFR has the lowest Si/Al ratio, which is due to the high Al_2_O_3_ content in RM. Some studies have pointed out that [SiO_2_(OH)_2_]^2−^ and [SiO(OH)_3_]^−^ will quickly combine with [Al(OH)_4_]^−^ monomers to form oligomeric aluminosilicates in geopolymers with a lower Si/Al ratio, which is not conducive to the development of strength [39]. Moreover, too low a Si/Al ratio results in a low concentration of [SiO(OH)_3_]^−^ monomers, which is not conducive to the formation of a three-dimensional network structure [40]. 

As can be seen, there is an obvious difference between the microstructure of the sample FSR and other samples, and the microstructure of the sample FSR is loose and porous as a whole. There are relatively less hydration products, and they cannot effectively fill the pores of the matrix. There are still some relatively intact FA and SS particles in the matrix, and the connection between the particles and the hydration products is weak, with obvious corners and boundaries, so the structure formed is disordered and low density [41]. The microstructure of other samples is relatively dense, and the generated cementitious material encapsulates most of the unreacted particles to form a relatively complete whole, indicating that Ca(OH)_2_ has optimized the structure of the matrix. In addition, the sample CFSR is denser and more complete than the sample CFR. This is because, on the one hand, the incorporation of SS can produce more hydration products in the matrix. On the other hand, SS, RM, and FA particles with different particle sizes form a close packing state with the hydration products, filling the pores of the matrix, and thereby improving the internal structure of the matrix. These results are consistent with the results of XRD, FTIR, TG-DTG, and MIP analysis.

## 4. Conclusions

In this work, the effects of Ca(OH)_2_ and SS on the mechanical properties and hydration products of FA-RM geopolymers were studied. The conclusions are as follows:

1. Both SS substitution of partial RM and Ca(OH)_2_ incorporation increased the compressive strength of the matrix at all ages. When one of the factors is considered separately, the incorporation of Ca(OH)_2_ is more obvious than that of SS to replace part of the RM to improve the mechanical properties of the FA-RM geopolymer. When SS replaces 50% RM and 4% Ca(OH)_2_ is added, the 28 d compressive strength of the prepared geopolymer material reaches 30.6 MPa.
2. The results of XRD, FTIR, TG-DTG, and SEM-EDS analysis demonstrated that both SS replacing part of the RM and Ca(OH)_2_ incorporation could promote the formation of more C(N)-A-S-H and C-S-H gels in the FA-based polymer matrix, as well as optimize the pore structure and reduce the porosity, which could significantly increase the compressive strength of the matrix.

## Figures and Tables

**Table 1:** The mix proportions of mortars (g). (SS: steel slag, RM: red mud, FA: fly ash).

Samples	SS	RM	FA	NaOH Pellet	Sodium Silicate	Ca(OH)_2_	Sand	Water
FR	0	225	225	18.83	141.49	0	1350	79.91
FSR	112.5	112.5	225	18.83	141.49	0	1350	79.91
CFR	0	225	225	18.83	141.49	18	1350	79.91
CFSR	112.5	112.5	225	18.83	141.49	18	1350	79.91

FR: Samples prepared using FA and RM as precursors without Ca(OH)_2_ incorporation; FSR: Samples prepared with FA, SS, and RM as precursors without Ca(OH)_2_ incorporation; CFR: Samples prepared with FA and RM as precursors and doped with Ca(OH)_2_; CFSR: Samples prepared with FA, SS and RM as precursors and doped with Ca(OH)_2_.

**Table 2:** Chemical compositions of raw materials (wt.%).

Binder	SiO_2_	Al_2_O_3_	Fe_2_O_3_	MgO	CaO	Na_2_O	K_2_O	SO_3_	TiO_2_	MnO	Other
FA	48.47	21.11	8.40	2.11	9.15	0.74	0.98	\	0.81	0.09	8.14
RM	13.49	20.39	30.58	0.32	15.82	9.15	0.19	0.66	7.80	\	1.6
SS	26.95	5.66	24.15	5.33	29.67	0.09	0.08	\	0.57	5.39	2.11

**Table 3:** Pore structure coefficient of different samples after curing for 28 d.

Samples	Total Pore Volume (mL/g)	Average Pore Diameter (nm)	Porosity (%)	Pore Size Distribution (%)		
				<20 nm	20–50 nm	>50 nm
FSR	0.31	37.23	42.82	10.63	48.79	40.58
CFR	0.29	19.07	41.98	39.72	29.99	30.29
CFSR	0.25	14.99	39.52	48.67	38.18	13.15

## Data Availability

Data are contained within the article.
